# Correction: Liu et al. Genetic Association Analysis of Skin Traits and the *TAF11* Gene in Shenxian Pigs. *Animals* 2026, *16*, 593

**DOI:** 10.3390/ani16081184

**Published:** 2026-04-13

**Authors:** Songzan Liu, Yu Li, Mingxin Sun, Wenjun Wang, Chunlian Lu, Hongzhan Cao

**Affiliations:** College of Animal Science and Technology, Hebei Agricultural University, Baoding 071001, China; liusongzan@163.com (S.L.); ly18731456994@163.com (Y.L.); 19932747941@163.com (M.S.); wangwenjun0807@163.com (W.W.)

**Author Order Change**

The order of the authors in the manuscript [1] is not correct.

An adjustment should be made to the physical order of the co-first authors as follows: from the original order of Yu Li, Songzan Liu to Songzan Liu, Yu Li. 

The authors state that the scientific conclusions are unaffected. This correction was approved by the Academic Editor. The original publication has also been updated.
